# Resource utilization for chimeric antigen receptor T cell therapy versus autologous hematopoietic cell transplantation in patients with B cell lymphoma

**DOI:** 10.1007/s00277-022-04881-0

**Published:** 2022-06-27

**Authors:** Alexander Ring, Björn Grob, Erik Aerts, Katharina Ritter, Jörk Volbracht, Bettina Schär, Michael Greiling, Antonia M. S. Müller

**Affiliations:** 1 Department of Medical Oncology and Hematology, University Hospital Zurich, Zurich, Switzerland; 2 Health Economics Market Access Pricing, Zurich, Switzerland; 3 Division of Controlling and Data Management, University Hospital Zurich, Zurich, Switzerland; 4 Institute for Workflow-Management in Health Care, European University of Applied Sciences, Berlin, Germany; 5 Department of Blood Group Serology and Transfusion Medicine, Medical University of Vienna, Währinger Gürtel 18-20, 1090 Vienna, Austria

**Keywords:** Chimeric antigen receptor T cells (CAR-T), Autologous stem cell transplantation (ASCT), Aggressive B cell lymphoma, Health care resource consumption, Comparative cost analysis

## Abstract

CD19-directed chimeric antigen receptor T cells (CAR-T) have emerged as a highly efficacious treatment for patients with relapsed/refractory (r/r) B cell lymphoma (BCL). The value of CAR-T for these patients is indisputable, but one-off production costs are high, and little is known about the ancillary resource consumption associated with CAR-T treatment. Here, we compared the resource use and costs of CAR-T treatment with high-dose chemotherapy followed by autologous stem cell transplantation (ASCT) for patients with r/r BCL. Standard operating procedures were used to develop a process model in ClipMed^PPM^, which comprises all activities and processes to sustain or generate treatment components that together constitute a treatment path. The software allows a graphic representation and the use of standardized linguistic elements for comparison of different treatment paths. Detailed processes involved in CAR-T treatments (*n* = 1041 processes) and in ASCT (*n* = 1535) were analyzed for time consumption of treatment phases and personnel. Process costs were calculated using financial controlling data. CAR-T treatment required ~ 30% less staff time than ASCT (primarily nursing staff) due to fewer chemotherapy cycles, less outpatient visits, and shorter hospital stays. For CAR-T, production costs were ~ 8 × higher, but overall treatment time was shorter compared with ASCT (30 vs 48 days), and direct labor and overhead costs were 40% and 10% lower, respectively. Excluding high product costs, CAR-T uses fewer hospital resources than ASCT for r/r BCL. Fewer hospital days for CAR-T compared to ASCT treatment and the conservation of hospital resources are beneficial to patients and the healthcare system.

## Introduction

Chimeric antigen receptor T cells (CAR-T) directed against CD19 have recently entered the clinic as a cellular treatment option for patients with relapsed/refractory (r/r) B cell malignancies. In Europe, the first commercially available CAR-T product, tisagenlecleucel (tisa-cel; CTL019; Kymriah®), was approved in 2018 by the European Medicines Evaluation Agency (EMEA) based on the pivotal phase II JULIET and ELIANA trials for patients with r/r diffuse large B cell lymphoma (DLBCL) and patients ≤ 25 years of age with r/r acute B-lymphoblastic leukemia (B-ALL), respectively [1, 2]. Shortly after, axicabtagene ciloleucel (axi-cel, Yescarta®) was approved for patients with r/r DLBCL and primary mediastinal large B cell lymphoma (PMBCL) based on the results of the ZUMA-1 trial [3]. In 2021, three additional CAR-T cell products have been approved by the FDA: Lisocabtagene maraleucel (liso-cel; Breyanzi®) for r/r large BCL (TRANSCEND trial) [4]; brexucabtagene autoleucel (KTE-X19; Tecartus®) for r/r mantle cell lymphoma (MCL) (ZUMA-2 trial) [5]; and idecabtagene vicleucel (ide-cel; Abecma®) for r/r multiple myeloma (KarMMA) [6]. The latter two, KTE-X19 and ide-cel, have also been approved in Europe in 2021.

Patients with r/r B cell lymphoma (BCL) who respond to 2nd-line salvage chemotherapy are considered chemo-sensitive and, if eligible, proceed to consolidation with high-dose chemotherapy and autologous stem cell transplantation (ASCT). Currently, commercially available CAR-T products are approved and offered to those who have failed at least two lines of therapy, are ineligible for high-dose chemotherapy with ASCT, or present with relapsed disease post ASCT. These patients have a very unfavorable prognosis, but there is the hope that CAR-T cells have a curative potential. In the registration trials, best overall and complete response rates in heavily pre-treated r/r B cell lymphoma (BCL) patients reached up to 83% and 58%, respectively[2–4]. Real-world experiences on response and outcomes, such as reports from the US CAR-T consortium [7], or the Spanish GELTAMO Group [8], resemble those of the phase II studies, with 1-year OS and PFS of 40–93% and 40–87%, respectively. Accordingly, there is a remarkable increase in the use of CAR-T cellular therapies reported to the registry of the European Society of Blood and Marrow Transplantation (EBMT) from only 151 patients in 2017 to 1134 in 2019 [9]. These numbers will continue to surge over the next years with the approval of more commercial CAR-T products, and a broader spectrum of treatment indications. Moreover, first results from randomized phase III trials were able to demonstrate the superiority of CAR-T as compared with ASCT for patients with aggressive BCL with refractory disease or early relapse post 1st-line immunochemotherapy [10], indicating that CAR-T cells may replace ASCT as a 2nd-line treatment for some patients in the near future.

Considering the rapidly increasing use of CAR-T cells, concerns are raised over the affordability of these highly expensive cell products. Several cost-effectiveness studies in the USA and Europe have discussed the value of these innovative treatment options in recent publications [11, 12]. Indeed, peer-reviewed studies have reported cost-effectiveness of tisa-cel and axi-cel in comparison with standard of care in the US healthcare system as well as in some European countries [12–17]. Such studies focus primarily on the societal cost–benefit tradeoffs, and less on individual patients and their healthcare providers.

Evidently, the one-off production cost of CAR-T is high. However, little is known about the ancillary resource consumption at the site of care (time, labor, materials) associated with real-world CAR-T therapy. Without a quantitative and detailed activity-based health economic assessment of CAR-T therapy, costs and resource use remain intangible and vague. Here, we model treatment components and processes involved in CAR-T treatment at our hospital against the current standard treatment for patients with r/r BCL (salvage chemotherapy followed by high-dose chemotherapy with ASCT) and compare the resource utilization and costs associated with these two treatment modalities. In contrast to previously published cost-effectiveness analyses, we present a highly transparent and granular assessment of activity-based resource consumption measured not only in financial units but also in time and personnel required for distinct processes, treatment components, and treatment paths. Our analysis shows that, apart from the high product costs, treatment with CAR-T cells bears the potential to save a substantial amount of hospital resources compared to standard therapy in patients with r/r BCL.

## Methods

### Research design and data gathering

For this study, we performed a detailed and comprehensive process analysis of r/r BCL patients undergoing CAR-T therapy or ASCT. Two distinct treatment paths for CAR-T and ASCT in patients with r/r BCL were mapped, modeled and analyzed. In our experience, r/r BCL patients for both treatment paths share many clinical features besides the underlying diagnosis, particularly as more than 50% of potential ASCT candidates will not respond to chemotherapy sufficiently and subsequently become eligible for CAR-T treatment [18].

Using standard operating procedures (SOP) in place at the University Hospital Zurich, we established detailed pre-models and models of patient scenarios. We modeled only scenarios of patients (i) that were treated at our hospital for the entire duration of their treatment; (ii) without any severe complications, including no transfer to the intensive care unit (ICU). As cellular therapies (i.e., ASCT and CAR-T) are audited and certified by JACIE and national authorities routinely, clear and comprehensive SOPs, checklists, and ancillary documents are available for each distinct procedure. Relevant documents were selected by physicians, nurses, and coordinators. Information on procedures was extracted from various sources (SOP, data management, controlling data/bookkeeping, and interviews with involved staff) for the time period from March 1, 2020 to November 30, 2020. Healthcare-related data for both treatment paths were used to define specific treatment components and processes for further analysis. We included different parameters to perform calculations on costs and expenditure of time, specifically (i) detailed time allocation by treatment phases and staff groups; (ii) total time; (iii) cost categories (materials/medications, personnel, hospital overhead and general costs); and (iv) total treatment costs.

### Software-based procedural health economic analysis

**Fig. 1:**
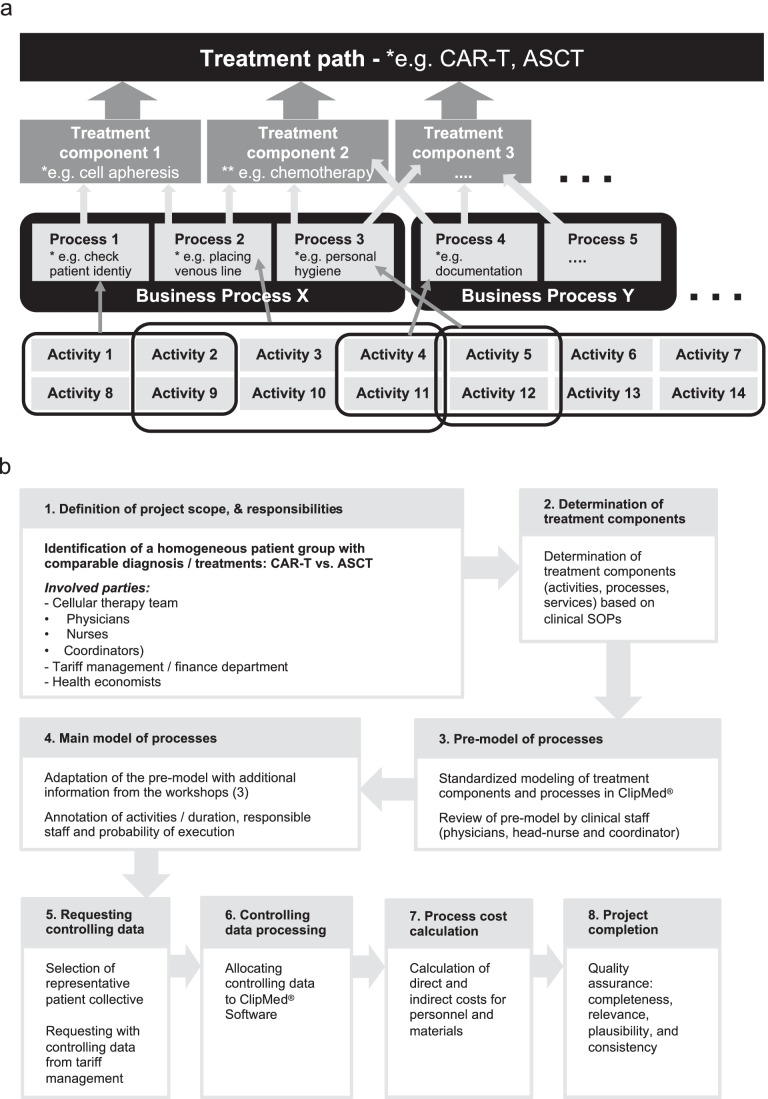
**a** Process-oriented modeling of treatment paths. A treatment path comprises a range of treatment components. A treatment component consists of processes, which are the sum of activities (= individual work steps). Business processes comprise a group of processes that are typically accounted for as one position for financial controlling. **b** Flow chart of project design and conduct of the analysis

The software-based procedural health economic analysis (SPHA) comprises detailed process-oriented modeling of the defined treatment paths (specifically CAR-T vs. ASCT in this project) and the respective accounting of process costs [19–22]. In such a “process-oriented model,” services provided by the hospital during a treatment path are identified. As displayed in Fig. 1a, “treatment components” are services such as “apheresis,” or “chemotherapy,” which include several individual “processes.” On a downstream-level, processes consist of “activities” (work steps) that together sustain or generate a process (activities processes treatment component treatment path). Processes, on the other hand, can be grouped into so-called “business processes,” which aggregates a distinct set of processes that typically are performed together and accounted as such regarding financial controlling.

The process-oriented model was created in ClipMed^PPM^, a well-established software tool that allows a graphic representation and the use of standardized linguistic elements to enable a comparison of different treatments. Additionally, the software calculates the costs of the treatment paths based on (i) hourly rate of involved staff; (ii) time needed to execute specific processes; (iii) the probability that a specific process occurs in the treatment path; and (iv) the direct material costs assigned to a process, such as medication and disposables. Surcharge costs, which cannot be directly allocated to specific processes, such as hospital overhead, infrastructure, laboratory costs, and general ward costs were distributed through a proportional allocation key. Process cost accounting, as performed by ClipMed^PPM^, is a full cost calculation method based on cost-by-cause principle of direct and indirect costs [23].

### Validation by extraction of primary controlling data

The findings from the process analysis and cost calculations were validated by comparing them with anonymized primary hospital controlling data and costs for CAR-T and ASCT treatment as generated within the hospital. Quality assurance was based on four main criteria: completeness, relevance, plausibility, and consistency. Interpretation of the results was done in collaboration with health care economic experts and medical personnel including nursing staff, coordinators, and physicians directly involved in the treatment procedures.

### Compliance with ethics guidelines

This work is based on SOPs, financial data, and hospital staff expert interviews and does not involve human participants.

## Results

### Definitions and process-oriented modeling

Here, we performed a detailed and comprehensive analysis of activities, processes, and treatment components that r/r BCL patients undergo within CAR-T treatment or high-dose chemotherapy with ASCT. For this analysis, we first defined the project scope and identified and documented responsibilities of the involved parties (Fig. 1b_1). Based on SOPs used in the clinic and staff interviews treatment paths, treatment components, and processes were dissected to a high level of granularity (“activities”/individual work steps; Fig. 1b_2) and entered into ClipMed^PPM^ software to generate a process-oriented pre-model of standardized treatment components (Fig. 1b_3). In several workshops this pre-model was thoroughly reviewed by involved clinical staff (physicians, nursing staff, transplant coordinator), adapted if necessary, and verified for correctness and coherence. All activities and procedures were parameterized with an amount of time (in minutes) and annotated with a description of the responsibilities of a given activity or procedure, also considering the probability that procedures and activities were performed (or not) (Fig. 1b_4). The model was subsequently applied for detailed analyses and cost-by-cause calculations. Using this main model a representative clinical patient collective was selected to request controlling data from the hospital’s billing unit (tariff management) (Fig. 1b_5). Processing controlling data on the ClipMed^PPM^ platform by allocation of tariff positions to clinical procedures allowed for calculations of direct and indirect costs for personnel and materials (Fig. 1b_6 + 7). Finally, to complete the project, quality assurance was based on the four criteria completeness, relevance, plausibility, and consistency (Fig. 1b_8).

### Comparison of time and resource expenditure of CAR-T versus ASCT

**Fig. 2:**
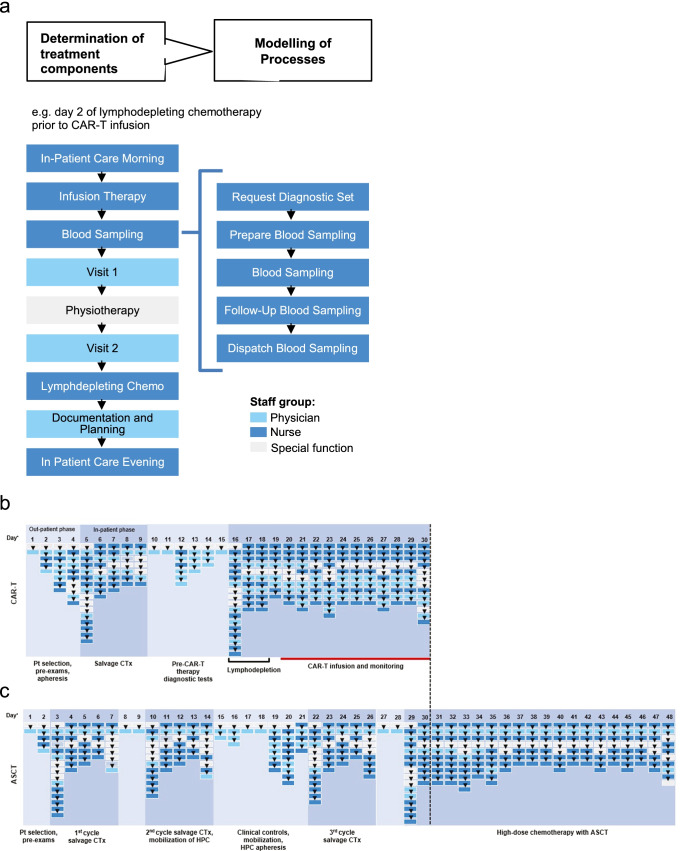
Detailed overview of individual processes per day during CAR-T (**a**) and ASCT (**b**) treatment: Each icon represents a treatment component, each color represents a staff group (light blue – physicians; dark blue — nursing staff; grey — special functions. *Displayed are only actual treatment days, treatment-pauses not considered

We determined treatment components and distinct processes for each individual day of treatment. Figure 2a displays an example of a regular inpatient day on which chemotherapy is administered and illustrates how a treatment component can be subdivided into processes. Exemplified is the treatment component “blood sampling” which involves distinct processes: (1) placing the order of diagnostic parameters; (2) preparation of equipment for blood draw; (3) blood draw; (4) post blood draw care; (5) sending blood samples to laboratory; and finally (6) assessment of results. For each activity duration, probability of execution, consumed materials, and medications were recorded.

- Treatment components provided by the hospital for the two distinct treatment paths based on SOPs and staff interviews are displayed in Fig. 2b for CAR-T and Fig. 2c for ASCT. Only days of activities in the hospital were considered and displayed, while days at home without consultations or exams were not part of the analysis. The CAR-T treatment path (tisa-cel or axi-cel) involved outpatient visits for evaluation of the eligibility for CAR-T, patient education regarding the treatment and risk management, securing reimbursement of treatment, 1 cycle of bridging chemotherapy (R-ICE or R-DHAP; given in the inpatient setting), pre-CAR-T check-up (with PET-CT, cMRI, pulmonary function test, echocardiography, EEG etc.), and hospitalization for CAR-T treatment with lymphodepletion (3 days of chemotherapy with fludarabine and cyclophosphamide), infusion of CAR-T and 10-day follow-up. For CAR-T therapy, all procedures and inclusion criteria were in accordance with approval criteria defined by the manufacturers and authorities.
- The ASCT treatment path involved outpatient visits for evaluation of transplant-eligibility, patient education for treatment, 3 cycles of salvage chemotherapy (R-ICE or R-DHAP), pre-ASCT check-up (with PET-CT, pulmonary function test, echocardiography etc.), and hospitalization for high-dose chemotherapy (BEAM (carmustine, etoposide, cytarabine, melphalan)) and ASCT.

Overall, CAR-T treatment involved on average 30 days of hospital care (inpatient and outpatient setting) vs. 48 days for ASCT (Fig. 2b and 2c, respectively). Importantly, the data presented provide an overview of the cumulative treatment time, which does not represent the total treatment duration as days on which activities were performed were not consecutive and recovery time and waiting periods were not counted. For CAR-T treatment we identified a total of 1041 individual processes (Fig. 2b) as compared with 1535 processes for ASCT (Fig. 2c), which is equivalent to a 32.3% reduction in processes for CAR-T therapy. For each day similar levels of detail were recorded (32 vs. 34.7 processes per day for CAR-T and ASCT, respectively) for both treatment paths. The slightly higher value per day for ASCT can be explained by the higher proportion of days of in-patient care (hospitalization time 73% for ASCT vs. 67% of treatment days for CAR-T), as a day of inpatient care generally involves more treatment components and processes than outpatient care.

### *Time* of direct health care is shorter in CAR-T versus ASCT treatment

**Fig. 3:**
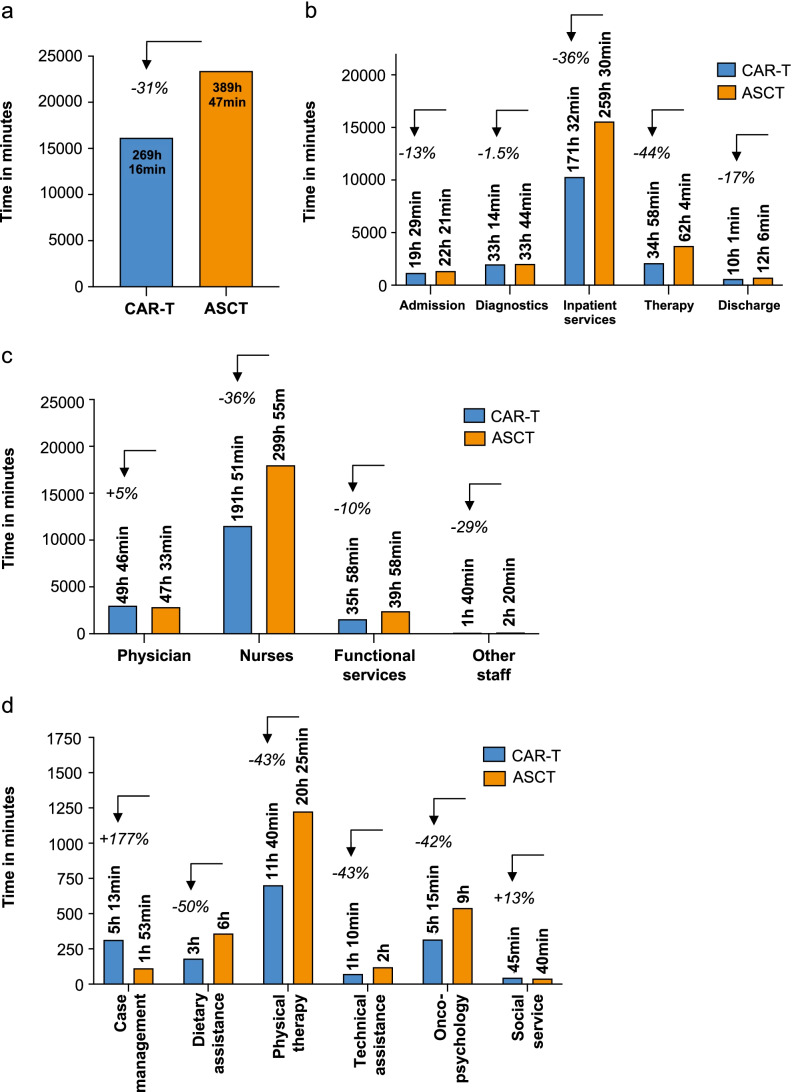
Time expenditure for CAR-T and ASCT. **a** Total time expenditure. **b** Time expenditure per business process. **c** Time expenditure per staff group. **d** Time expenditure per functional service

Total time for CAR-T treatment was 269 h and 16 min compared with 389 h and 47 min for ASCT, equivalent to a 31% reduction in overall treatment time for CAR-T compared to ASCT (Fig. 3a). We next analyzed the dedication of time to distinct categories of processes (“business processes”) related to patient admission, diagnostic procedures, inpatient services, pharmacological therapies, and discharge (Fig. 3b). The category *admission* includes administrative tasks such as setting up the appointment, preparation of the appointment, and the actual patient appointment, as well as additional activities by medical staff (e.g., taking patient history, planning of diagnostic/therapeutic procedures, and documentation). *Diagnostics* include laboratory testing (e.g., ordering diagnostic tests, blood draws, distribution of blood samples to various labs, electro encephalogram (EEG), echocardiography, ECG, cMRT, PET-CT, pulmonary function test, and bone marrow biopsies (in 20% of cases) plus the interpretation of results. *Inpatient services* include mainly nursing activities (e.g., vital signs, personal hygiene, administering medication, organizing meals) and physician activities (placing and removing catheters, rounds, documentation etc.). The category *therapies* includes preparation and administration of chemotherapies, apheresis, cell re-infusion, and documentation of these procedures. *Discharge* involves the following components: physician discharge (final examination, discharge letters, prescriptions), nursing discharge (discharge instructions and documentation), and organization of follow-up care. The discharge procedure also includes patient registration in national registries, which is mandatory for cellular therapies (Swiss Blood Stem Cell; SBST, the Swiss registry feeds into the EBMT database).

Comparing both treatment paths the main difference observed was related to *inpatient services* and *therapies* due to the shorter treatment duration of the CAR-T therapy (30 days vs. 48 days) and less therapeutic interventions (i.e., 3 cycles of salvage therapy for ASCT vs. 1 cycle of bridging therapy for CAR-T). As displayed in Fig. 3b, the overall reduction of 31% in cumulative treatment time for CAR-T as compared with ASCT can be further dissected into a 36% reduction of time dedicated to inpatient services (171 h 32 min vs. 259 h 30 min); a 44% reduction of time spent on therapeutic procedures and interventions (34 h 58 min vs. 62 h 4 min); and a 13% (19 h 29 min vs. 22 h 21 min) and 17% (10 h 1 min vs. 12 h 6 min) reduction of time used for procedures associated with patient admission and discharge, respectively. No difference was observed for diagnostics for CAR-T and ASCT treatment paths.

We next analyzed time expenditure per staff group. The greatest difference between the CAR-T and ASCT paths were observed for nurses, which had a reduction of 108.1 h (36%) per patient over the entire treatment period (Fig. 3c). As both therapies require intensive nursing time (71.2% of total CAR-T treatment time vs. 76.9% for ASCT), this difference represents a considerable advantage for CAR-T therapy over ASCT in personnel resource-constrained hospital environments. Overall, nurses spent 4–6 times more time with patients compared with physicians. For physicians, the cumulative expenditure of time was slightly higher (+ 5%) for CAR-T treatment than for ASCT, which can be explained by higher upfront investment in administrative tasks (including discussions with insurance companies), but also more time spent at the bedside monitoring toxicities and side effects following CAR-T infusion (Fig. 3c). Approximately 36 and 40 h of functional services (physical therapy, onco-psychological support, case management, dietary assistance, social work, technical assistance (e.g., thawing of cell products)) were performed with each CAR-T and ASCT patient, respectively (− 10% for CAR-T vs. ASCT) (Fig. 3d). As expected, case management and coordination required more time for CAR-T than for ASCT treatment (+ 177%), likely due to the novelty and complexity of CAR-T related tasks (including tariff management; interactions with insurance companies etc., administrative matters, logistics of cell pick-up and delivery). In contrast, a reduction in time was observed for CAR-T in repeatedly occurring services (i.e., physical therapy 8.75 h (− 43%), psycho-oncological care 3.75 h (-42%)), which can be attributed to the overall shorter treatment duration. Hence, time requirements for services that occur only once or twice during a hospital stay (i.e., dietary assistance (here at the beginning and end of an inpatient stay), social services (usually once towards the end of hospital stay)) were similar for both treatment paths. For technical assistance (freezing and thawing of stem- and CAR-T cells), it is noteworthy that CAR-T are mostly contained in one single bag compared with 2–5 infusion bags for ASCT, which reduces thawing and re-transfusion time. Moreover, for axi-cel, the patient’s cells (as starting material) are shipped fresh, which saves additional staff time required for freezing in the hospital’s cell laboratory. CAR-T cell treatment was associated with an overall 43% time reduction for technical procedures.

**Fig. 4:**
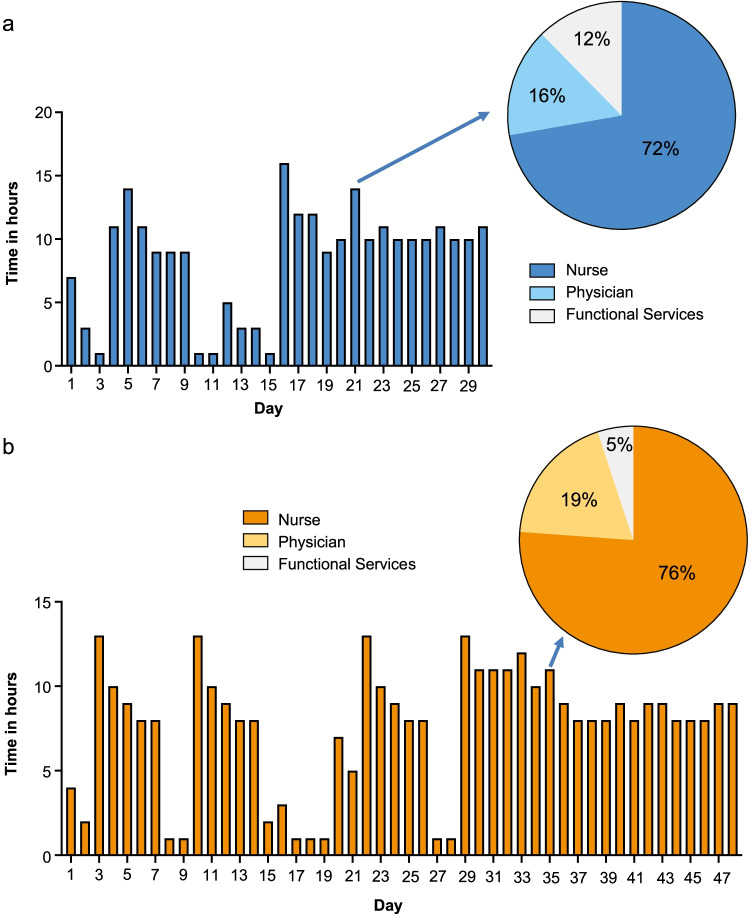
Cumulative treatment time per day for CAR-T (**a**) and ASCT (**b**). The figures display the hours of treatment per treatment days, including — in more detail — the exemplary day of cell infusion (day 21 for CAR-T (**a**); day 35 for ASCT (**b**)). Total cumulative treatment time for CAR-T was 30 days and for ASCT 48 days

Figure 4 displays time (hours) expenditure per treatment day (non-consecutive days), for each treatment path. The insets illustrate time allocation per staff group (i.e., nurses, physicians, functional services) on day 21 and day 35, which represent the re-transfusion days for CAR-T (Fig. 4A) and ASCT (Fig. 4B), respectively.

### CAR-T total cost is higher compared with ASCT, but more cost-effective when excluding upfront production costs

**Fig. 5:**
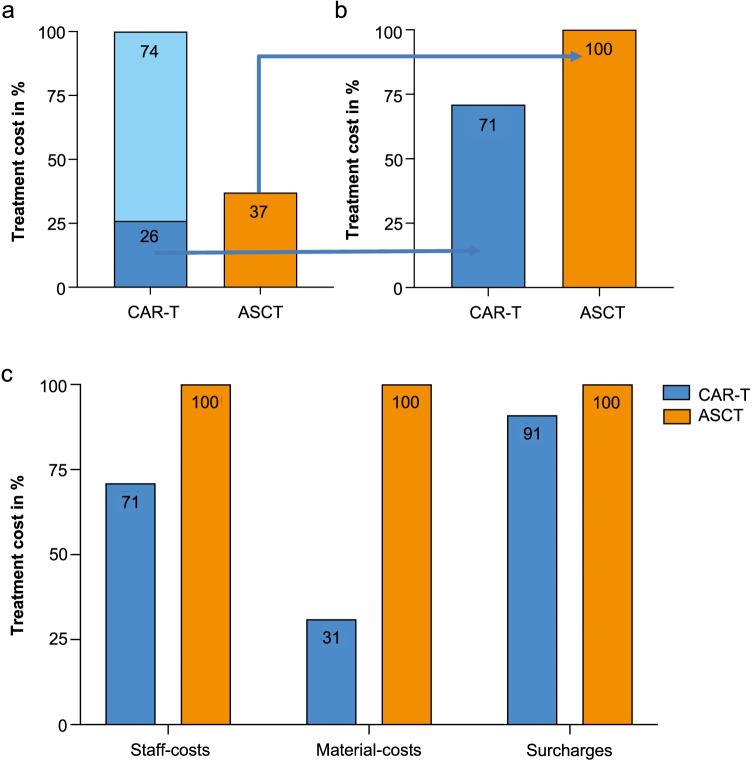
Relative treatment costs CAR-T vs. ASCT. **a** Total treatment costs, including production of CAR-T, were 63% higher, for CAR-T vs. ASCT. **b** Treatment cost excluding CAR-T production expenditure for CAR-T for CAR-T vs. ASCT. **c** Treatment costs, excluding CAR-T production cost, split into personnel, material cost, and surcharges

Total treatment costs were higher for CAR-T compared to ASCT due to the one-time production cost (+ 63% for CAR-T) (Fig. 5a). However, when calculating treatment costs excluding the CAR-T product (which accounted for 74% of the total costs), the costs for CAR-T were 29% lower (Fig. 5b). For a more granular picture, we analyzed and divided non-CAR-T product associated costs into different categories: (i) Staff costs were calculated using average salaries for physicians and nurses and accounted for 20% of the total costs in both treatment paths. With shorter duration of overall treatment the total costs for staff salaries were 29% lower for CAR-T as compared with ASCT (Fig. 5c). Material costs included mainly expenses for drugs (e.g., chemotherapy, supportive medications), and some disposables (e.g., catheters) and represented 27% of total costs for ASCT, while only 12% of total costs in CAR-T patients, thereby being 69% higher for ASCT. The higher share of material costs in ASCT can be explained by additional chemotherapies and to a lesser extent by supportive co-medications during the longer inpatient period. The analysis also incorporated surcharge costs of other involved medical departments that cannot be directly allocated to a patient, such as costs related to laboratory, cardiology, radiology and nuclear medicine, infrastructure, and overhead (53% of total ASCT costs vs. 68% of total CAR-T costs, excluding production). The higher share of surcharge costs for CAR-T is the consequence of the more extensive diagnostic workup. Yet, overall, surcharge costs were 9% lower for CAR-T compared with ASCT (Fig. 5c).

## Discussion

We present a comprehensive process-oriented analysis comparing CAR-T with high-dose chemotherapy and ASCT treatment for patient with r/r BCL using single-center data at a Swiss University Hospital, based on time and cost requirements for both treatments. We used a multidisciplinary approach for data acquisition and well-established software-based (ClipMed^PPM^) methodology. While the significantly higher upfront cost for CAR-T treatment is generally well known, our analysis yielded several major findings that paint a more nuanced picture of health care resource utilization associated with CAR-T and standard of care treatment (ASCT).

Excluding the high upfront costs for manufacturing of the gene-modified cellular CAR-T product, our analysis illustrates that CAR-T therapy can be carried out with less hospital resources and less cumulative time investment than ASCT (30 vs 48 days for CAR-T and ASCT, respectively). In general, both treatments require substantial resources of the nursing staff, which binds > 70% of the cumulative treatment time (CAR-T: 71%, ASCT: 77%). Thus, by reducing the total treatment duration with CAR-T approximately 100 h (36%) less per patient are needed for nursing care compared with ASCT. Due to the higher logistic complexity of CAR-T, including prolonged discussions with insurance companies for reimbursement and diagnostic procedures pre- and post-CAR-T infusion, the absolute hours invested by physicians were similar between both procedures, despite shorter total treatment time for CAR-T therapy. Time expenditure was also higher for case managers in the case of CAR-T treatment, which we attributed to more complex logistics (i.e., ordering cells on company-specific online-platforms, shipping cells to the manufacturer, planning of treatment and procedures around the anticipated date of delivery of the CAR-T product) compared with ASCT (i.e., in-house freezing and storage of autografts). It is conceivable that these processes become less extensive and less time consuming as the use of CAR-T cells becomes more routine. Our analysis also illustrates how administrative activities impact the profession of physicians by shifting their activities away from medical procedures, which comprised < 10% of the total process time for both treatment paths.

Estimates on the overall cost of CAR-T therapy for all hematological cancers range between 11.1 billion EUR (12.5 billion USD) and 88.8 billion EUR (100 billion USD) for the period from 2019 to 2029 [24, 25]. Such high costs present a major challenge to the health care system and to society. However, CAR-T and other innovative therapies, including other forms of cell and gene therapies, appear to cause a paradigm shift in treatment, with curative potential and hence long-term benefit for patients and society. Recent evidence from large phase III randomized trials suggests the superiority of CAR-T over ASCT in certain clinical contexts (NCT03570892, NCT03391466, NCT03575351) [26–28]. The ZUMA-7 study (NCT03391466) demonstrated superiority of axi-cel over ASCT in patients with early relapse following induction therapy, and so did the TRANSFORM study (NCT03575351) for liso-cel, while the BELINDA study (NCT03570892) was not able to proof superiority of tisa-cel over ASCT. However, patient selection, bridging strategies, and intensity of lymphodepleting chemotherapy were different across the trials, and therefore no direct comparison of the efficacy of the available CAR-T products is legitimate before head-to-head studies are performed. Yet, the studies show, that depending on patient selection, bridging strategies, timing of CAR-T treatment, and other factors to be determined, for distinct patient groups CAR-T cells, bear the potential to replace ASCT to increase overall outcomes.

T cells from less heavily pre-treated patients provide better starting material for in vitro generation of CAR-T [29, 30], and earlier CAR-T treatment will reduce therapy associated toxicities and costs of prior treatment lines. Improvements in disease biomarker, patient selection, and in vivo immunomodulation may add to this development and ultimately further improve outcomes. For many patients, replacing ASCT with CAR-T cells would embody a major achievement, reducing aggressiveness and duration of the treatment while possibly improving outcomes. Finally, as our results show, hospital resources may be preserved and should be considered, especially in times of severe nursing staff shortages.

Medical centers providing CAR-T therapy in Switzerland are currently faced with standardized DRG-based reimbursement criteria reflecting “standard of care” treatments. Alternative reimbursement schemes are needed to calculate the “true” cost of CAR-T, incorporating the benefits described above: coverage with evidence development schemes, negotiated outcomes-based staged payment agreements, outcomes-based rebates [1, 2]. In the light of recent approvals of CAR-T products for additional disease entities and indications, including plasma cell myeloma, in which the high costs of the CAR-T product need to be compared with years of continuous novel triple and quadruple treatments, studies like ours are of high relevance and provide transparency and detailed information to encourage discussions about efficiency and optimization of processes and resource utilization for increased treatment quality.

Other strengths of our study are the multidisciplinary approach and well-established methodology, which provides insight into resources and costs for a distinct clinical scenario, as well as the cost structure of treatment days, organizational units and treatment phases. In contrast, most conventional calculation methods calculate costs using standard surcharge rates (e.g., days of care), which do not reveal causal relationships between procedures, resources, and costs. This transparency and level of detail should be of considerable valuable for other institutions and across borders, since medical procedures and non-financial resource consumption are likely similar for most treatment components of cellular therapies, which are highly regulated and standardized by JACIE/FACT.

Limitations of our study include the single-center and “theoretical” nature of the study design. We deliberately excluded scenarios of patients with serious side effects both for CAR-T and ASCT for several reasons: (1) ICU stays were rare and their duration was highly variable, ranging from 24 h to several weeks. ICU stays occurred both in patients with ASCT and CAR-T treatment. Given the small case number and the more theoretical nature of the study, using standard operating procedures, no meaningful comparison could be derived. (2) In order to provide a simplified benchmarking system within and between institutions, we decided to exclude complications with highly variable duration and costs, not only due to the reason stated under (1), but also because of lack for ICU standards for CAR-T complications. Nevertheless, rigorous evaluation of resource consumption related to adverse events involving ICU treatment is urgently required in future studies. Similarly, since no post-CAR-T re-admissions related to CAR-T toxicities occurred in our program, and no specific standard operating procedures exist for such scenarios, we excluded this setting from our study, too. Thus, since policies, procedures, and also outcomes might be different in other institutions or in future scenarios, our results should be interpreted carefully and with appropriate adaptions wherever indicated. In contrast to other analyses on the cost-effectiveness of CAR-T cell therapy that derive value from incremental survival and health-related quality of life benefit [11, 12, 31], we did not perform economic evaluations or budget impact analyses to investigate the economic burden of new therapies to the society and health care system. We also did not consider patient outcomes into the analysis by measurement of so-called quality adjusted life years (QUALYs) per dollar spent [12]. An important caveat of our analysis is that we only provide a theoretical framework with potential applicability to institutions like our own. Data input producing specific results will have to be adapted for each institution.

Since the bulk of health economic studies consider cost aspects, we add to the published literature valuable information about saving of time resources as a critical advantage of CAR-T therapies, besides their clinical benefits. Our study provides a basis for an objective, data-based discussion for hospital resource management optimization, with benefits for both patients and health care providers.

Overall, excluding high product costs, CAR-T uses strikingly fewer hospital resources than standard of care treatment (ASCT). These saved time resources can be used to resource planning, organization of health care facilities, and ultimately improve quality and safety in hospitals that are faced with growing challenges to ensure adequate care (increasing shortage of skilled personnel, high number of beds with complex patients, and expensive maximum care) [32, 33]. But perhaps even more importantly shorter duration of therapy will increase precious quality lifetime for patients [34, 35].

## Declarations

### Data availability

Primary data are available on request from bgrob@hemap.ch.

### Code availability

ClipMed PPM Version 4.4.
